# The diversity of zinc-finger genes on human chromosome 19 provides an evolutionary mechanism for defense against inherited endogenous retroviruses

**DOI:** 10.1038/cdd.2013.150

**Published:** 2013-10-25

**Authors:** S Lukic, J-C Nicolas, A J Levine

**Affiliations:** 1 Simons Center for Systems Biology, Institute for Advanced Study, Einstein Drive, Princeton, NJ 08540, USA

**Keywords:** Zinc-fingers, retrotransposons, germline

## Abstract

Endogenous retroviruses (ERVs) are remnants of ancient retroviral infections of the germ line that can remain capable of replication within the host genome. In the soma, DNA methylation and repressive chromatin keep the majority of this parasitic DNA transcriptionally silent. However, it is unclear how the host organism adapts to recognize and silence novel invading retroviruses that enter the germ line. Krueppel-Associated Box (KRAB)-associated protein 1 (KAP1) is a transcriptional regulatory factor that drives the epigenetic repression of many different loci in mammalian genomes. Here, we use published experimental data to provide evidence that human KAP1 is recruited to endogenous retroviral DNA by KRAB-containing zinc-finger transcription factors (TFs). Many of these zinc-finger genes exist in clusters associated with human chromosome 19. We demonstrate that these clusters are located at hotspots for copy number variation (CNV), generating a large and continuing diversity of zinc-finger TFs with new generations. These zinc-finger genes possess a wide variety of DNA binding affinities, but their role as transcriptional repressors is conserved. We also perform a computational study of the different ERVs that invaded the human genome during primate evolution. We find candidate zinc-finger repressors that arise in the genome for each ERV family that enters the genomes of primates. In particular, we show that those repressors that gained their binding affinity to retrovirus sequences at the same time as their targets invaded the human lineage are preferentially located on chromosome 19 (*P*-value: 3 × 10^−3^).

Transposable elements (TEs) are nucleotide sequences that are able to either change their relative position or to increase their copy number in a host genome. Almost 50% of the human genome consists of sequences derived from TEs. The large repertoire of molecular mechanisms that TEs employ to increase their copy number include: (1) cut-and-paste mechanisms mediated by transposases that are able to recognize the ends of the transposon in the host DNA and cut and insert them somewhere else in the host genome; (2) transcription of TE mRNA followed by the expression of endonucleases and reverse transcriptases that are able to integrate newly synthesized DNA copies of the retrotransposon into the host genome; and (3) retroviral-like mechanisms, in which some expressed endogenous retroviruses (ERVs) assemble virus particles to infect and insert their genomes into the chromosomes of germ cells (for more details see^1, 2^). Given that the mobilization of these elements to new locations in a genome has the potential to be deleterious to the host, it is expected that mechanisms have evolved in the host to counter the expansion of TEs.^3^

Recent progress has shed light on the mechanisms by which the host evolves *trans*-acting repressor elements that recognize *cis*-acting sequences in a retrotransposon, thereby blocking its expression. The goal of this paper is to understand how the host learns to identify and repress newly invading TEs by studying the evolution of a prominent system of repressors in the human lineage. We build on recent work that has pointed to the Krueppel-Associated Protein 1 (KAP1) and its binding partners as major contributors to the formation of repressive chromatin states on ERVs.^4, 5, 6, 7^ Although the current understanding of the molecular mechanisms by which KAP1 and its partners repress endogenous retrotransposons has grown in recent years (see Figure 1), the evolutionary mechanisms involved in the targeting of newly inherited ERVs are still poorly understood. In this study, we propose that particular genomic locations that sit on mutational hotspots are continuously generating new zinc-finger genes with unique DNA binding affinities. A subset of these zinc-finger genes are involved in the recognition and repression of inherited ERVs by partnering with KAP1. This mechanism allows the host to generate a variety of zinc-finger motifs permitting the possible recognition of a newly inherited ERV, even before the retrovirus has colonized the host genome.

The aim of this paper is to study how the zinc-finger proteins that recruit KAP1 to the genome evolve to recognize and target new inherited retroviruses. We first show evidence that KAP1 is recruited by zinc-finger proteins to sequences on human ERVs by analyzing a ChIP-seq data set for wild-type KAP1 and a mutant KAP1 in which the domain that binds to the zinc-finger proteins was deleted. Second, we study the evolution of the DNA-binding specificity of the associated zinc-finger genes in the evolutionary lineage of humans. We show how many of the zinc-finger genes sit in clusters associated with copy number variant (CNV) formation hotspots on human chromosome 19. In addition, we use a computational technique to predict the DNA binding sites of every human zinc-finger gene, to show how the zinc-finger genes that target sequences contained in ERV DNA are preferentially located on human chromosome 19.

Finally, in the discussion, we review other mechanisms of host recognition of transposable elements (TEs) that have been proposed in the literature. We argue in favor of a new model in which zinc-finger genes found in a continuous array on chromosome 19 undergo recombination generating CNVs and new genes whose proteins recognize novel DNA sequences, some of which are found in retrotransposons. Because those retrotransposons that are unchecked by recognition of a repressor zinc-finger can go on to kill the host, we expect to observe in present day organisms a good correspondence between zinc-finger genes that recognize and bind to those retrotransposons that have recently entered the human lineage (and survived). We observe that correspondence in the lineage of primates analyzed here. Only in a scenario in which the host population is producing a large reservoir of repressors with different DNA binding affinities do the offspring that inherit a new ERV have a significant chance to somatically silence it and reproduce at a reasonable rate.

## Results

### Human KZNF transcription factors recruit KAP1 to binding sites located on endogenous retroviral DNA and other TEs

To quantify the frequency with which human KAP1 is recruited to endogenous retroviral DNA, we analyzed a recently published ChIP-seq data set for KAP1.^8^ The authors determined the genomic location of KAP1 by means of chromatin immunoprecipitation of KAP1 followed by next-generation sequencing experiments performed on three different cell lines (human embryonic kidney 293 cells (HEK293), U2OS and K562 cells, see Materials and Methods). The authors found a total of 18 760 autosomal peaks spanning 8 900 411 base pairs. We annotated every repetitive DNA sequence contained in the peaks using RepeatMasker. Of the 8.9 Mbp spanning 18 760 autosomal peaks, 3.7 Mbp were annotated as repetitive DNA. In particular, 3.6 Mbp were annotated as TEs, which include long terminal repeat (LTR) elements, DNA transposons, long interspersed nuclear element retrotransposons (LINEs) and short interspersed nuclear element retrotransposon (SINEs). LTR elements alone, which include ERVs, spanned 1.64 Mbp of the 18 760 autosomal peaks. These 1.64 Mbp that are annotated as LTR elements span 18% of the binding sequences of KAP1. As these proportions depend on the particular chromatin states existing in the cell types, we also compared the relative abundances of TE-derived DNA in the regions of accessible chromatin of HEK293 and K562 cells with the relative abundances of TE-derived DNA in the binding peak sequences (see Table 1 and Materials and Methods). We observed that the binding of KAP1-associated TFs on LTR elements and LINEs is between fourfold and eightfold more frequent than expected in a null model of random binding.

**Figure 2:**
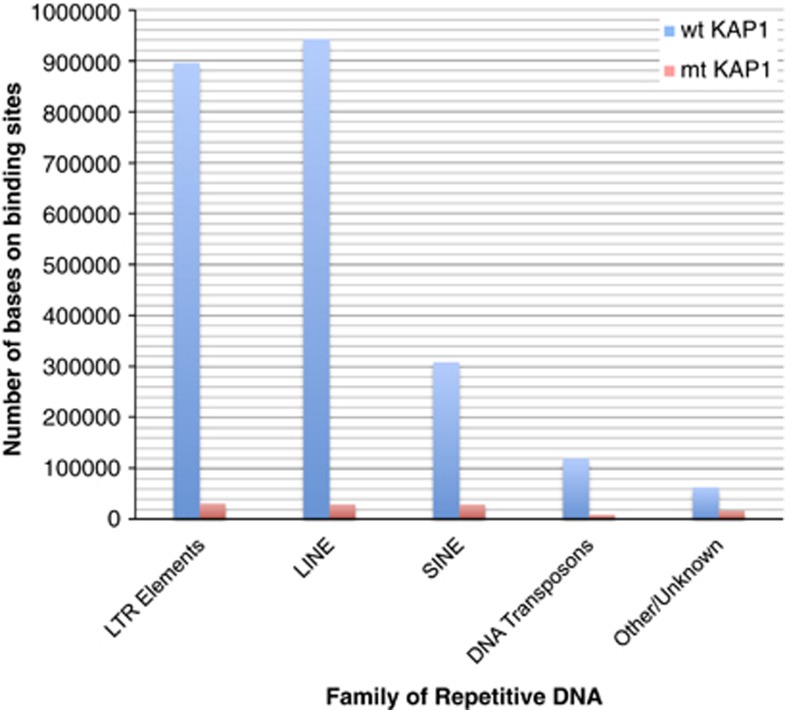
Abundance of binding DNA associated with mutant KAP1 (red) and wild-type KAP1 (blue) in different families of repetitive elements. Only ∼1% of DNA on binding sites annotated as TEs (LTR elements, LINE, SINE and DNA transposons) that was present in the ChIP-seq peaks associated with wt KAP1 was also present in the peaks associated with mt KAP1. The abundance of DNA-binding sequence was measured in units of millions of base pairs (Mbp)

The next question we explored was what factors that interact with KAP1 recognize this parasitic DNA? The available evidence demonstrates that the recruitment of KAP1 to endogenous retroviral DNA is mediated by the interaction of its RBCC domain with the Krueppel-Associated Box (KRAB) domain present in many TFs.^9^ To test this hypothesis, we compared the binding sites of a mutant KAP1 with no RBCC domain (mt KAP1) *versus* the binding sites of wild-type KAP1 (wt KAP1) on HEK293 cells^8^ (see Materials and Methods section). We inferred the binding peaks using the MACS algorithm.^10^ We applied a *P*-value cutoff of 10^−10^ and identified a total of 20 139 autosomal peaks for wt KAP1 and 732 autosomal peaks for mt KAP1. To reduce the fraction of misidentified peaks, we considered only the subset of peaks that had also been inferred previously. We observed a very large depletion of binding sites for the mutant KAP1-ΔRBCC (mt KAP1). In particular, mt KAP1 was found in only ∼4% of the binding sites on TE-coding DNA compared with experiments where wild-type KAP1 was used (wt KAP1) (see Figure 2). In the case of LTRs and LINEs, only ∼3.5% of the wt binding sites on LTR elements were present in the experiment with mt KAP1. Hence, this supports the hypothesis that KAP1 is recruited to endogenous retroviral DNA by KRAB-containing TFs. This observation is consistent with experiments on mouse cells, in which the knockout of KAP1 gave rise to the transcriptional derepression of LINEs and ERVs.^4^

Finally, to estimate the fraction of KRAB-containing TFs that use a tandem of zinc-fingers to bind to DNA, we downloaded the Superfamily database.^11^ We found a total of 793 KRAB domains on human genes. Of these, 753 KRAB domains were located on genes that have at least one zinc-finger motif; and, of those, 635 were located on genes explicitly annotated as zinc-finger genes in RefSeq. Therefore, we estimate that a minimum of 80% and a maximum of 95% of protein-coding KRAB domains are located in zinc-finger transcription factor (TF) genes. This supports a model in which KAP1 targets and silences TEs after being recruited by zinc-finger proteins.

### Clusters of ZNF genes on chromosome 19 are located in hotspots for CNV formation

We scanned the human genome (assembly GRCh37/hg19) so as to identify all of the C2H2 (cystine-2 histidine-2 amino-acid sequence motif present in a large family of zinc-finger proteins) zinc-finger protein motifs. The motif consists of six similar conserved sequences of between 21 and 25 amino acids. We found a total of 8080 non-overlapping C2H2 motifs on coding genes in the human genome. A total of 1854 different coding genes contained at least one C2H2 motif, whereas 748 genes contained at least one tandem of C2H2 zinc-fingers. Here, we defined a tandem of zinc-fingers as a set of two or more C2H2 motifs separated by less than 200 base pairs each. As tandems of zinc-finger domains (ZFs) evolve quickly under duplications and deletions, it is not possible to determine whether the ancestor of a given tandem belonged to a KRAB-containing TF or not. Because of this, in order to study the evolution of this quickly evolving class of genes, we considered every C2H2 ZF that is present in the human genome.

**Figure 3:**
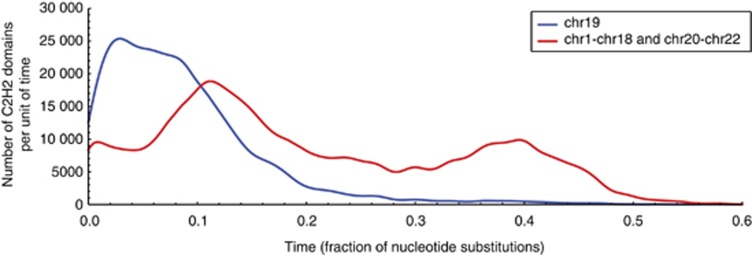
Age distribution of C2H2 ZFs in chromosome 19 (blue curve) and in the remainder of the autosomes (red curve). The area below each curve describes the total number of zinc-fingers in the corresponding time interval. The total number of C2H2 domains in chromosome 19 is 3513, whereas the remainder of the autosomes contain 4567 zinc-finger domains. The age of a particular C2H2 sequence is defined as the time of the most recent duplication event associated with such C2H2 domain. The duplication times are computed as the branch lengths of a phylogenetic tree and the units of time consist of the fraction of nucleotide substitutions (f.n.s.) (see ‘Analysis of C2H2 motifs in Materials and Methods'). The high density of zinc-fingers for times smaller than 0.2 f.n.s. in chromosome 19 denotes a recent burst of duplications of zinc-fingers on this chromosome. This contrasts with the expansion of C2H2 domains on the remainder of the autosomes which, although they span 98% of all autosomal DNA, they only contribute a third of all recent duplications of zinc-fingers. This huge expansion of C2H2 zinc-fingers in the human lineage is specific to chromosome 19 and probably occurred during mammalian evolution

In order to understand the evolutionary relationships between these C2H2 zinc-fingers, we computed the sequence similarity between every pair of C2H2 domains (see Materials and Methods and Supplementary Information). We used this to estimate the age of different duplications (low sequence divergence denotes recent events, whereas large sequence divergence corresponds to older events). We observed a significant number of C2H2 zinc-fingers in human chromosome 19 (see Figure 3). This gene expansion is associated with duplication events that have less than 10% of nucleotide substitutions (fairly recent expansions, see Figure 3). Using the chicken genome as an outgroup, we infer that this expansion is specific to mammals.^12^

**Figure 4:**
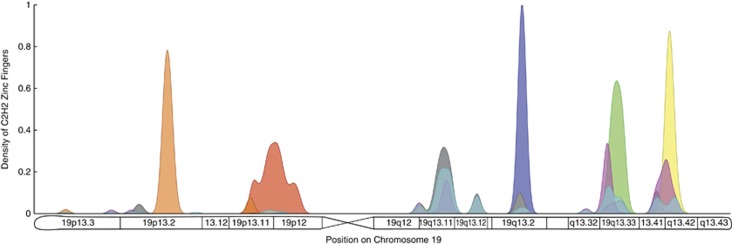
Distribution of similar C2H2 zinc-finger domains on chromosome 19. Each zinc-finger sequence was assigned to one of eight different clusters based on a hierarchical clustering analysis (see ‘Analysis of C2H2 motifs in Materials and Methods'). Each of these clusters is represented by a different color: orange, red, violet, green, yellow, gray, light blue and magenta. We used the sequence divergence between pairs of zinc-fingers as the similarity metric in this analysis. The density associated with each cluster was normalized such that the total mass is one, and it is represented with a particular color in the figure. We found that five clusters of zinc-fingers are highly localized on particular regions of chromosome 19 (orange, red, violet, green and yellow). This supports the hypothesis that local duplications generated by unequal crossover events have been responsible for the expansion of clusters of C2H2 zinc-fingers

We classified all the C2H2 zinc-fingers on chromosome 19 using their sequence similarity. We found that similar C2H2 sequences cluster together around one of six major clusters in chromosome 19 (see Figure 4 and Materials and Methods). We found a strong positive correlation between sequence divergence and physical distance. This is consistent with the hypothesis that the expansion of clusters on chromosome 19 has been driven by local duplications associated with unequal crossover events.

**Figure 5:**
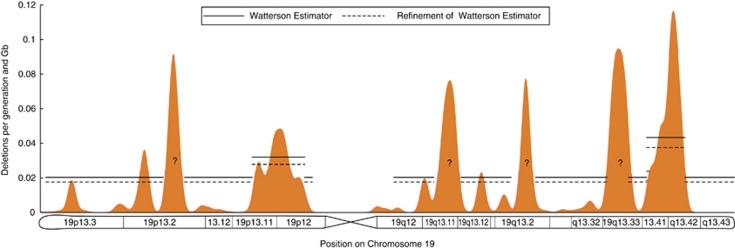
Deletion rate on chromosome 19. The orange probability density represents the empirical distribution of 3513 zinc-finger motifs on chromosome 19. The deletion rate was estimated using population frequency data of polymorphic deletions larger than 50 bp in 45 individuals of European ancestry (CEU from 1000 genomes project). We used the Watterson estimator (continuous horizontal line) and a refinement of the Watterson estimator (dashed horizontal line) built from the frequency spectrum of deletions (see ‘Deletion rate as a function of the genomic location' in Supplementary Information). We estimated the deletion rate on seven different regions of chromosome 19. These regions include six segments with a high density of zinc-finger motifs (peaks on 19p13.2, 19p13.11-19p.12, 19q13.11, 19q13.2, 19q13.33 and q13.41-q13.42) and the complementary region of chromosome 19. However, because of lack of sufficient data we could only estimate the rate on three segments: the complementary region, the peak on 19p13.11-19p.12 and the peak on q13.41-q13.42. The question marks in the plot denote the four segments where we could not estimate the deletion rate with confidence. The deletion rate is expressed in units of number of deletions (larger than 50 bp) per generation and per Giga-base (Gb)

An additional question that we explored is whether the expansion of clusters of zinc-fingers on human chromosome 19 is an ongoing phenomenon. To test this hypothesis, we estimated the current CNV formation rate along chromosome 19 using population genetics methods applied to human data (see Supplementary Information and Figure 5). Because it is not clear how to root CNVs with copy number larger than 2, the analysis was restricted to deletions. We applied two different estimators of the deletion rate (see Figure 5) to sequence data associated with 45 individuals of European ancestry.^13^ The inferred chromosome-wide deletion rate for deletions larger than 50 base pairs was 0.017–0.022 deletions per generation and per Giga-base. The deletion rate in only two of the six clustered regions with a high density of C2H2 zinc-fingers on chromosome 19 could be estimated because the other four regions did not have enough gene duplications to provide statistically significant results. Both estimators predicted that the deletion rate in the clusters located on 19p13.11-19p.12 and on 19q13.41-19q13.42 is about twofold higher than the background deletion rate.

In summary, our population genetics analysis of the CNV formation rate on clusters of zinc-fingers on chromosome 19 provides evidence for a twofold higher CNV mutation rate in some of the clusters when compared with the background rate across the genome.

### Zinc-Finger genes that gained their DNA binding affinity at the same time when their target ERV family invaded the human lineage are located on chromosome 19

In this subsection, we show evidence that supports the notion that the tandems of zinc-fingers that gained their binding affinities at the same time as their predicted target ERV family invaded the human lineage are preferentially located on chromosome 19 (see Table 2). We inferred this by means of a genome-wide analysis of the coevolution of tandems of zinc-fingers and ERVs in the primate phylogeny (see ‘Detection of Zinc-Finger domains and families of ERVs in primate and rodent genomes' in Supplementary Information) and the use of a Support Vector Machine (SVM) trained by Persikov *et al.*^14^ to predict the top candidate DNA binding sites of a given tandem of C2H2 zinc-finger protein (see ‘Prediction of DNA binding affinities associated with tandems of Zinc-Fingers' in the Supplementary Information).

We used 52 families of ERVs in the human genome that had been previously annotated as primate-specific by RepeatMasker. We isolated 4700 insertions of ERVs in the human genome representing these 52 different families (see Materials and Methods and Supplementary Information). By mapping the DNA sequences associated with these insertions to the different primate genomes, we were able to estimate the time at which each family invaded the human genome (see Materials and Methods and Supplementary Information). We obtained results that were similar to those reported in previous studies.^15, 16^ In addition, by comparing the binding affinities of tandems of homologous zinc-fingers across the phylogeny, we were able to estimate the time at which any given human tandem of zinc-fingers gained their current binding affinity. Finally, we used the SVM (see Materials and Methods) to predict the top candidate DNA binding sites on ERV genomes for every tandem of zinc-fingers in humans (see Table 3). We achieved this by searching for family-specific sequence motifs on ERVs that belonged to the predicted spectrum of most strongly bound states for a given tandem of zinc-fingers in a protein.

Using these results, we were able to restrict our analysis to repressors that obtained their binding affinity at about the same time when their target ERVs invaded the human lineage. Then, we compared the number of predicted repressors located on chromosome 19 with the number of predicted repressors located elsewhere (see Table 2 and section ‘Inference of the preferential location of zinc-finger repressors of ERVs on chromosome 19 using Fisher's exact test with a noisy classifie**r'** in Supplementary Information). Comparing this distribution with the background distribution of all tandems of ZF genes, we were able to conclude that the zinc-finger gene repressors were preferentially located on chromosome 19 when compared with the rest of the genome by a ratio of 7 : 2 zinc-finger gene repressors of ERVs. This supports the main thesis of this paper: CNV formation hotspots located at chromosome 19 have contributed to the generation of new TFs that have become repressors of new inherited ERVs.

## Discussion

Transposons have invaded the genomes of almost every eukaryotic organism that has been examined for this property. On the positive side, these insertions provide new gene sets that can be modified and adapted to new functions in the host and/or can modify transcriptional programs that may speed up evolutionary changes. However, these TEs also introduce a large number of deleterious alterations in the host genome. In particular, once a TE is inserted into the genome, its movement to new sites increases the probability for detrimental events. For instance, insertional mutagenesis can inactivate gene functions. The induction of novel transcriptional activities in the genomic region of integration sites can initiate cancers and abnormal developmental processes. In addition, multiple insertions of TEs create sites for homologous recombination and potential deletions. These detrimental effects exert selective pressures to remove the TEs (e.g., by recombination between LTR sequences),^16^ and to evolve mechanisms that prevent TEs from invading, or, once established, to prevent these TEs from expressing their functions and enhanced replication and movement.^15^ For instance, the PIWI RNAs are derived from antisense TE sequences that hybridize with the TE RNA in the germ line and the soma resulting in the degradation of the TE RNA.^17, 18, 19^ Both of these mechanisms, the purging of deleterious insertions of TEs by means of natural selection and the production of repressors using antisense sequences from the genome of the TE as a template, consist of host responses after the invasion of the TE. In general, this type of explanation has been the only alternative that is considered when reviewing the evolution of TEs and their repressors.^1^

In humans, about 8% of the human genome consists of remnants of ERV elements associated with several dozen independent invasions in the human lineage.^15^ More recently, additional viral sequences have been found integrated into a wide variety of eukaryotic genomes, indicating a large number of independent viral invasions during eukaryotic evolution.^17^ In this paper, we have presented evidence in favor of a hypothesis in which the host population can generate a diverse set of zinc-finger gene repressors, a subset of which can inactivate the expression of a newly inserted ERV; this permits both colonization of the new ERV and its control by the host. We have shown how the zinc-finger genes that transcriptionally silence ERV-coding sequences are located on clusters on chromosome 19 that form CNV formation hotspots. These clusters generate new combinations of zinc-finger genes via recombination providing sets of proteins, of which a subset is capable of recognizing novel retrovirus sequences. This is an efficient way to generate the needed diversity of zinc-finger genes to counter the diversity of TEs. These zinc-finger proteins act as repressors of transcription of the ERVs that are newly inserted into the host genome, thus minimizing the negative effects of these TEs. This model predicts that the generation of a zinc-finger gene *via* recombination will be fixed into the genome by the selective advantage of inactivating a TE insertion whose sequences are recognized by the zinc-finger protein. Thus, the evolutionary time scales of ERV insertions and the appearance of the zinc-finger gene that binds to its unique sequences should occur at the same time. When this idea was tested over the time scales of primate evolution, this appeared to be the case, and the zinc-finger genes that inactivate the TE are commonly located on chromosome 19. We find this model appealing, because the recurrence of independent invasions of ERVs can be countered by a reservoir of zinc-finger repressors that are continuously generated on CNV formation hotspots.

## Materials and Methods

### ChIP-seq data

We downloaded from the UCSC genome browser the table of Transcription Factors binding sites (Txn Factor ChIP on human genome assembly GRCh37/hg19) for KAP1. The data consisted of the ChIP-seq peaks called by the Sole-Search algorithm.^18^ The sequence reads were obtained from the combination of ChIP-seq experiments performed on three different cell lines (HEK293, U2OS and K562) and mapped to the human genome using the Illumina Genome Analyzer Pipeline.^8^ We found a total of 19 427 peaks genome wide. The average length of the peaks was 474 bp, the S.D. was 74 bp and the peaks in the 2.5th and 97.5th percentiles of the distribution of lengths had lengths 424 bp and 668bp, respectively. We annotated the repetitive DNA sequences on peaks by means of the RepeatMasker table available in the UCSC genome browser.

The sequence reads for the ChIP-seq experiments with wt KAP1 and mt KAP1 in HEK293 cells were downloaded from the NCBI GEO Data Sets web page under the accession numbers GSM700353 and GSM700355.^8^ To characterize the open chromatin regions in HEK293 and K562 cells, we used the tables wgEncodeOpenChromDnaseHek293tPk and wgEncodeOpenChromDnaseK562Pk from the genome.ucsc.edu/ENCODE, consisting of DNAse I hypersensitive sites.^19, 20^

### Analysis of C2H2 motifs

We translated the autosomal part of the human genome (human genome assembly GRCh37/hg19) on both strand orientations and for every possible codon combination in search of C2H2 motifs. We studied the evolutionary relationship of protein-coding C2H2 sequences by means of the Unweighted Pair Group Method with Arithmetic Mean (UPGMA) phylogenetic tree and the Jukes–Cantor approximation (see Supplementary Information S.I.).

### Detection of ZFs and families of ERVs in primate and rodent genomes

We scanned six primate genomes in addition to the human genome to find tandems of zinc-fingers that are homologous to those of humans. This allowed us to estimate the time at which the DNA binding affinity of any given tandem was first established (see Supplementary Information for more details on this).

## Figures and Tables

**Figure 1:**
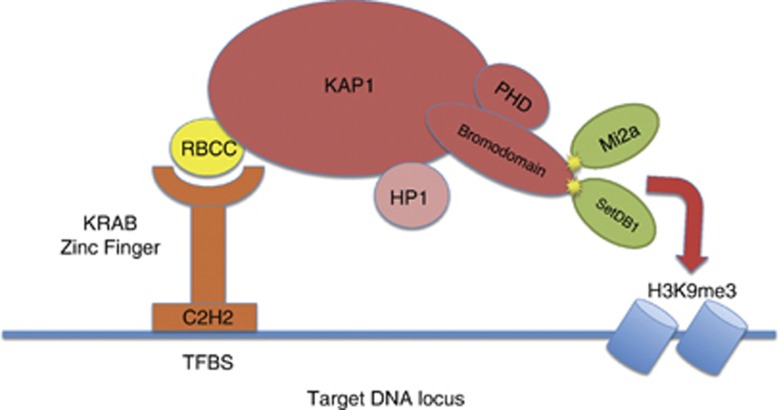
KAP1 has an N-terminal tripartite motif (TRIM) containing an RBCC domain (ring finger, two B-box zinc-fingers and a coiled coil), a central HP1 (heterochromatin protein 1) domain and a C-terminal combination plant homeodomain (PHD) and bromodomain (B). These three domains have been shown to mediate nuclear localization, interaction with TFs, oligomerization and regulation of transcription.^21^ The RBCC domain interacts with the KRAB module present in the KRAB-C2H2 zinc-finger proteins (KZNF). In addition to the RBCC domain, every other subdomain of KAP1 contributes to the remodeling of chromatin on genomic loci targeted by the KRAB-containing TFs.^9^ For instance, the HP1-binding domain (PxVxL) interacts with HP1 family members, whereas the KAP1–HP1 complex has a role in silencing euchromatic and pericentric heterochromatic regions.^21^ The PHD and bromodomain interact with two chromatin-modifying enzymes: Mi2a and SETDB1, of which SETDB1 encodes a histone methyltransferase involved in histone methylation, gene silencing and transcriptional repression^8^

**Table 1:** Comparison of the abundance of TE-derived DNA in the regions of accessible chromatin of two cell types with the abundance of the same TE sequences that are contained in the binding peaks associated with KAP1

**Family of elements**	**HEK293 cells**		**K562 cells**	
	**Fraction of accessible chromatin** (**%**)	**Fraction of binding peaks for KAP1** (**%**)	**Fraction of accessible chromatin** (**%**)	**Fraction of binding peaks for KAP1** (**%**)
LTR elements	3.9	16.9	5.4	31.2
LINEs	3.8	12.0	4.2	32.8
SINEs	3.0	4.2	3.7	3.3
DNA transposons	1.2	1.6	1.3	1.04

Abbreviations: KAP1, KRAB-associated protein 1; KRAB, Kruppel-associated box; LINEs, long interspersed nuclear element retrotransposons; LTR, long terminal repeat; SINEs, short interspersed nuclear element retrotransposon

The data are shown for HEK293 cells and K562 cells. The abundance of DNA is measured in units of base pairs. Both data sets show how KAP1 preferentially targets either LTR elements or LINEs but not SINEs or DNA transposons

**Table 2:** Contingency table for the genome-wide distribution of tandems of fingers predicted to repress particular ERV families

	**ZNF tandems on chromosome 19**	**ZNF tandems on other chromosomes**
ERV-binding	32	9
Binding to any sequence	2492	1898
Fraction of ERV-binding tandems	1.3%	0.47%

Abbreviation: ERV, endogenous retrovirus

The number of repressive tandems located on human chromosome 19 is compared with the number of other predicted repressive tandems. In addition, the distribution of the number of predicted repressors is compared with the distribution of all the (not necessarily repressive) tandems of zinc-fingers. A significant bias for the repressive tandems to be located in chromosome 19 can be inferred if one compares the location of the repressive tandems with the location of all the (repressive and not repressive) tandems (*P*-value: 3 × 10^−3^, see section ‘Inference of the preferential location of zinc-finger repressors of ERVs on chromosome 19 using Fisher's exact test with a noisy classifier' in Supplementary Information). This supports our general hypothesis that the expansion of tandems of fingers on chromosome 19 contributed to the generation of repressors against new invading ERVs during primate evolution

**Table 3:** Most significant ZF genes predicted to repress particular endogenous retroviral families in the human genome

**ERV family**	**Zinc-Finger genes predicted to have tandems that strongly bind motifs in the ERV family**
HERV17-int	ZNF486*^,#^, ZNF74^#^, ZNF420^#^, ZNF717^#^
LTR19-int	ZNF621*^,#^, ZNF833P, ZNF729^#^, ZNF433*^,#^
PRIMA4-int	ZNF107*, ZNF479^#^, LOC643955, ZFP64
PABL_B-int	ZNF345*, ZNF845^#^, LOC100293516^#^, ZNF546^#^
HERVK22-int	ZNF100*^,#^, ZNF222^#^, ZNF7^#^, ZNF416^#^
HERVFH21-int	ZNF845*^,#^, ZNF16, ZNF627^#^, ZNF565^#^
LTR57-int	ZNF502*, ZNF268^#^, ZNF761, ZNF560^#^
HERVIP10FH-int	ZNF471*^,#^, ZNF615^#^, ZNF57^#^, ZNF780B*^,#^
HERVS71-int	ZNF726*^,#^, ZNF629, ZNF273^#^, ZFP57^#^
HERVK11-int	ZNF497*, ZNF79^#^, ZNF337^#^, ZNF658B*^,#^
HERVH-int	ZNF419*^,#^, ZNF460^#^, ZNF320^#^, ZNF700*^,#^
HERVL18-int	ZNF549*^,#^, ZNF736^#^, ZNF337^#^, ZNF528*^,#^
HERV4_I-int	ZNF585B*^,#^, ZNF883, ZNF721^#^, ZNF568^#^
LTR23-int	ZNF146*
HERVK3-int	ZNF729*^,#^, ZNF195^#^, ZNF432^#^, ZNF613^#^
HERVL66-int	ZNF490*^,#^, ZNF727^#^, ZNF41, ZNF823^#^
LTR46-int	ZNF682*^,#^, ZNF550^#^, ZNF540^#^, ZNF729^#^
HERVK13-int	ZNF586*^,#^, ZNF253*^,#^, ZNF551*^,#^, ZNF625-ZNF20*^,#^
HERV1_I-int	ZNF404*^,#^, ZNF81, ZNF136^#^, ZNF586*^,#^
MER84-int	ZNF568*^,#^, ZNF525^#^, ZNF132^#^, ZNF738^#^
HERVL-int	ZNF266*^,#^, ZNF41, ZNF879^#^, ZNF415*^,#^
HERVI-int	ZNF772*^,#^, ZNF502, ZNF57^#^, ZNF30*^,#^
HERVH48-int	ZNF571*^,#^, ZNF419*^,#^, ZNF442^#^, ZNF416^#^
HERVE_a-int	ZNF600*, ZNF626*^,#^, ZNF254^#^, ZNF223^#^
MER61-int	ZNF10*^,#^, ZNF594, ZNF34*^,#^, ZNF490^#^
HERV30-int	ZNF813*^,#^, ZNF789^#^, ZNF154^#^, ZNF675*^,#^
HERV9-int	ZNF230*^,#^, ZNF433^#^, ZNF619^#^, ZNF709^#^
HERV-Fc1-int	ZNF727*^,#^, ZNF772^#^, ZNF181^#^, ZNF343^#^
HERV15-int	ZNF726*^,#^, LOC100293516^#^, ZNF548^#^, ZNF107*
PABL_A-int	ZNF70*, ZNF729^#^, ZNF526, ZNF680*^,#^
HERVP71A-int	ZNF850*^,#^, ZNF737^#^, ZNF699^#^, ZNF765^#^
HERVK14C-int	ZNF253*^,#^, ZNF197^#^, ZNF251^#^, ZNF738*^,#^
HERVFH19-int	ZNF225*^,#^, ZNF491, ZNF273*^,#^, ZNF497
HERVIP10F-int	ZNF471*^,#^, ZNF57^#^, ZNF599^#^, ZNF718^#^
HERV3-int	ZNF14*^,#^, ZNF418^#^, ZNF727^#^, ZNF643^#^
HERVKC4-int	ZNF678*^,#^, ZNF718^#^, ZNF445^#^, ZNF211^#^
LTR25-int	ZNF34*^,#^, ZNF680^#^, ZNF446^#^
HERVE-int	ZNF611*^,#^, ZNF135^#^, ZNF525^#^, ZNF107
MER4-int	ZNF133*^,#^, ZNF43^#^, ZNF337^#^, ZNF529*
HUERS-P2-int	ZNF528*^,#^, ZNF780B^#^, ZNF491, ZNF2^#^
PRIMAX-int	ZNF416*^,#^, ZNF729^#^, ZNF345^#^, ZNF484*^,#^
HERVK14-int	ZNF621^#^, ZNF585B, ZNF208^#^, ZNF317^#^
HERVK11D-int	ZNF490^#^, ZNF667^#^, ZNF585B^#^, ZNF490^#^
HUERS-P3-int	ZNF676^#^
HUERS-P1-int	ZNF197^#^, ZNF543^#^, LOC100379224^#^, ZNF101^#^
HERVK-int	ZNF708^#^, ZNF847P, ZNF836^#^, ZNF673
LTR43-int	ZNF614^#^, ZNF843, ZNF443^#^, ZNF132^#^
MER101-int	ZNF837, ZNF268^#^, ZNF621^#^, ZNF721^#^
PRIMA41-int	ZNF611^#^, ZNF83^#^, ZNF526, ZNF438
HERVK9-int	ZNF788^#^, ZNF16, ZNF709^#^, ZNF333^#^
MER41-int	ZNF568^#^, ZNF470^#^

Abbreviations: ERV, endogenous retrovirus; KRAB, Kruppel-Associated Box; ZF, zinc-finger domain

The predictions are based on the DNA binding affinity of tandems of fingers for particular ERV-specific family-defining DNA motifs (see ‘Prediction of DNA binding affinities associated with tandems of Zinc-Fingers' of the Supplementary Information ). The number of predictions for some ERV families is low or absent whenever few or no tandems of fingers yielded significant scores for binding to those families. We used an asterisk (*) to denote those ZF genes that gained their DNA binding affinity at the same time when their predicted ERV targets invaded the human lineage. In addition, we use a pound sign (^#^) to denote those ZF genes that contain a KRAB sequence motif

## Abbreviations

C2H2: cystine-2 histidine-2 amino-acid sequence motif present in a large family of zinc-finger proteins
CNV: copy number variant
ERV: endogenous retrovirus
HEK293: human embryonic kidney 293 cells
K562: immortalized myelogenous leukemia cells
KAP1: KRAB-associated protein-1
KRAB: Krueppel-associated box transcriptional repressor domain
LINE: long interspersed nuclear element retrotransposon
LTR: long terminal repeat
RBCC: amino-acid sequence motif consisting of a RING, a B-box type 1 and a B-box type 2, and a coiled-coil region
SINE: short interspersed nuclear element retrotransposon
SVM: support vector machine
TE: transposable element
TF: transcription factor
TRIM28: tripartite motif-containing 28
U2OS: human osteosarcoma cell line
ZF: zinc-finger domain
