# Supplementary material for: The diversity of zinc-finger genes on human chromosome 19 provides an evolutionary mechanism for defense against inherited endogenous retroviruses
Source: Cell Death Differ. 2013 Oct 25;21(3):381–7. doi: 10.1038/cdd.2013.150 (PMC3921586; doi:10.1038/cdd.2013.150)
Supplement: Supplementary Information [file cdd2013150x1.doc]

Supplementary Information for: The diversity of zinc finger genes on human chromosome 19 provides an evolutionary mechanism of defense against inherited endogenous retroviruses.

Authors: Sergio Lukic, Jean-Claude Nicolas, Arnold J. Levine

**Deletion rate as a function of the genomic location**

We estimated the genomic map for the deletion rate in the European population using publicly available genome-wide sequence data from individuals with northern European ancestry (CEU) (1). The subset of the data that we used consisted of 10,742 deletions (larger than or equal to 50 base pairs) genotyped at low coverage in 45 CEU individuals. Also, we used the set of 7,186,258 Single Nucleotide Polymorphisms (SNPs) genotyped in 60 CEU individuals to estimate demographic parameters. We filtered the data by using only those deletions that had been called in more than 15 individuals and SNPs that had been called in more than 40 individuals. We restricted our analysis to variants located on chromosome 19. This left a total of 139 polymorphic deletions and 133,946 SNPs on chromosome 19.

We divided chromosome 19 into seven DNA segments. Six of these segments were associated with regions of high density of Zinc Fingers, while the remaining segment consisted of the complementary region of DNA on chromosome 19 (see Figure 5 of the main text). For each segment, we computed the Allele Frequency Spectrum (AFS) of deletions associated with a sample of 20 chromosomes (as the number of chromosomes was not constant and depended on the particular genotyped deletion, we projected down to 20 chromosomes). We rooted the AFS by defining the ancestral state as that which has copy number two (cn=2). States with smaller copy number were assumed to be derived after the occurrence of a past deletion event.

We estimated the deletion rate by means of summary statistics of the frequency spectrum of polymorphic deletions. In particular, we used the Watterson estimator


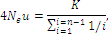


where n=20 is the number of chromosomes sampled, K is the total number of bi-allelic polymorphisms in the sample (SNPs or deletions),
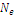
 is the effective population size and u is the mutation rate. K can also be interpreted as the total number of polymorphisms in an AFS with n chromosomes. As in Gravel et al. (2) we assumed a divergence time of 6 million years between human and chimpanzee, a generation time of 25 years, and we estimated a point mutation rate of
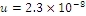
 mutations per base and per generation. We used the point mutation rate
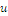
 and the observed AFS of SNPs to estimate
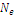
, which we used to estimate the deletion rate
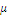
 given the observed AFS of polymorphic deletions. Additionally, we applied the error model introduced in (2) to correct for low-sequencing errors in the observed AFS for SNPs. Using SNPs on chromosome 19, we inferred that the effective population size in CEU is
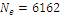
.

As the Watterson estimator makes use of some rigid assumptions (e.g. mutation-drift equilibrium), we also estimated the deletion rate using a more sophisticated diffusion method in population genetics (3) (4). In particular, we used a non-equilibrium diffusion model that incorporates the out-of-Africa population bottleneck and the influx of de novo deletions associated with the CEU population. The corresponding forward diffusion equations can be written as


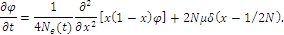


Here,
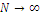
 denotes the *census population size*, and the time dependence of the *effective population size* is modeled as
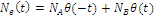
, with
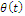
 the step function,
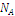
 the ancestral population size for t<0 and
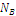
 the population size after the out-of-Africa event. We use a time clock in which t=0 denotes the time of the migration out of Africa and t=T>0 denotes the present time. We solved the Partial Differential Equation (PDE) using numerical methods (4) and fitted these models by means of the likelihood function associated with a Random Poisson Field.

In our application to estimate the deletion rate,
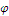
 denotes the density on frequency space of deletions contained in a DNA segment (with
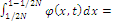
total number of polymorphic deletions in the population), and
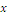
 denotes the frequency of a deletion. As we assume Hardy-Weinberg equilibrium and the genotypes of a deletion are classified by the copy number (cn) in each individual genome, the frequencies of each genotype are:
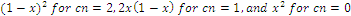
. Hence, given a set of demographic parameters, the expected number of polymorphic deletions
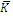
in a sample of size n=20 is


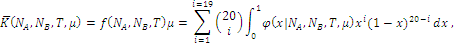


where the function
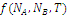
 can be evaluated as
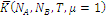
. This equation allows us to estimate the deletion rate by inferring first the demographic parameters
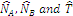
, using the SNP frequency spectrum and the associated point mutation rate
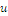
. Then, one can estimate the deletion rate on a genomic locus using the inferred values for
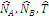
 and the observed number of polymorphic deletions
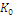
 in the locus as


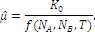


We denote this estimator as a *refinement of the Watterson estimator* (see Figure 5), because in the steady state limit (
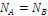
) one recovers the standard Watterson estimator.

**Zinc-Finger domains and families of endogenous retroviruses in primate and rodent genomes**

***Evolution of Zinc Finger genes in the human genome:***

We found a total of 55,855 non-overlapping candidate C2H2 motifs in the human genome, of which 8,080 C2H2 were located on protein coding genes annotated in RefSeq. We indexed each of the 8,080 C2H2 sequences on coding exons by means of a positive integer number *i* from 1 to 8,080. We denote by s_i_ the *i^th^* C2H2 sequence. We computed the similarity metric d(s_i_,s_j_) between every pair of sequences s_i_ and s_j_, using the Jukes-Cantor approximation


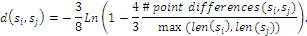


Here, len(s_i_) and len(s_j_) are the respective numbers of base pairs in the sequences s_i_ and s_j_, and #differences(s_i_,s_j_) consists of the number of nucleotide differences (base substitutions and indels) that exist in the best alignment of s_i_ and s_i_. We aligned each pair of sequences at the level of tri-nucleotides such that the 2 Cs, the 12 amino acids between C and H, and the 2 Hs were always aligned. When the number of unspecified amino acids between the two Cs or the two Hs was not the same in s_i_ and s_j_, we constructed the best alignment by looking every possible way to eliminate the excess of tri-nucleotides from the longer sequence to match the shorter one. In this way, we evaluated d(s_i_,s_j_) for every possible pair of sequences (i.e. more than 32.6 million pairs).

As we only consider the divergence between pairs of paralogous C2H2 domains within the same species, it is reasonable to assume a constant rate of evolution accordingly with the UPGMA algorithm. We estimated the age of a given C2H2 as the time of the most recent duplication event associated with this C2H2 domain. The duplication times were computed as the corresponding branch lengths in the UPGMA phylogenetic tree. The unit of time is the fraction of nucleotide substitutions.

Finally, we clustered C2H2 sequences by means of their sequence similarity d(s_i_,s_j_). To that end, we used the UPGMA phylogenetic tree as a method of hierarchical clustering with the distance metric d(s_i_,s_j_) defined above. We used the computer implementation available in the python package **hcluster** (<http://code.google.com/p/scipy-cluster/>).

***Zinc Finger genes in other animal genomes:***

We used six primate genomes in addition to the human genome to study the evolution of zinc finger genes. We used the mouse genome as an outgroup for primates. We downloaded the genomes from the Ensemble and UCSC genome browser webpages. In particular, we used the Mouse (NCBI37/mm9, 2007), Tarsier (tarSyr1, 2010 update), Marmoset (WUGSC 3.2/calJac3, 2009), Rhesus macaque (rheMac2, 2006), Orangutan (WUGSC 2.0.2/ponAbe2, 2007), Gorilla (gorGor3.1, 2011) and Chimpanzee genomes (CGSC 2.1.3/panTro3, 2010).

Although all of the fingers share the consensus sequence C-X_2,4_-C-X_12_-H-X_3,4,5_-H, we found that 712 of the 748 human genes that contain tandems of zinc fingers consisted only of the two shorter versions of the motif (C-X_2_-C-X_12_-H-X_3_-H and C-X_2_-C-X_12_-H-X_4_-H). This represents more than 95% of all genes with tandems of C2H2 motifs. Therefore, for simplicity, we restricted our detection of zinc fingers to the two shorter versions of the Cys2His2 motif. As the quality of the annotations of some of these genomes was very poor, we translated every genome on both strand orientations and for every possible codon combination in search of C2H2 motifs. We found 31,749 C2H2 motifs in mm9, 23,105 in tarSyr1, 23,941 in calJac3, 23,038 in rheMac2, 27,291 in ponAbe2, 23,934 in gorGor3 and 25,478 in panTro3.

We computed the sequence divergence between pairs of zinc fingers to find candidate homologous tandems in mouse and other primates. In particular, given a tandem of fingers in human, we searched for those tandems in every other species that had the smallest sequence divergence. As sometimes there were several candidate homologous tandems, we always kept the three closest tandems in each species. For a given tandem of fingers in human, we estimated the time when the current DNA binding affinity was established by comparing the DNA binding affinities of every other homologous tandem. As the vast majority of the base-specific contacts in the zinc finger–DNA complexes are made from the positions −1, 2, 3, and 6 of the α-helix (5), we estimated the DNA binding affinity of a tandem of fingers by reading the -1, 2, 3 and 6 amino acids in each finger and using the Support Vector Machine trained by Persikov et al. (6) to compute its spectrum of bound states (see subsection **Prediction of DNA binding affinities associated with tandems of Zinc-Fingers**). As we had three candidate homologous tandems per species (other than human) and per tandem in human, we selected the tandem that had the most similar spectrum of bound states to the human tandem. In this way, we estimated the time when a given human tandem gained its current DNA binding affinity as the branch of the phylogenetic tree that separates two consecutive species in which a significant change in the DNA binding affinity first occurred. We denoted this branch by the names of the two consecutive species that are connected in the phylogenetic tree. We only considered those tandems in which any other species that shared older common ancestors with human also had a significantly different spectrum of bound states to that of the homologous human tandem.

Also, we isolated 4,700 insertions of primate-specific endogenous retroviruses (ERVs) in the human genome with the goal of identifying particular retroviral sequences that belonged to the spectrum of bound states of some tandem of zinc fingers. We used RepeatMasker to identify every family of LTR elements in the human genome that had been classified as primate-specific in previous studies (e.g. see (7) (8)). In particular, we used a set of LTR elements that consisted of 52 different families of ERVs (8).

As previous experimental studies had identified the binding site of the KRAB zinc fingers close to the Primer Binding Site (PBS) of the virus (9), we searched for all the retroviral insertions that contained an intact copy of the 5’ UTR. In particular, we scanned the whole human genome for primate-specific insertions that contained the 5’ LTR plus a minimum of 100 base pairs on the adjacent viral genome. This constraint gave rise to 4,700 different insertions representing the 52 different families of ERVs. In addition, we used BLAST (2.2.25) to map the 4,700 sequences to the six different primate genomes and to the mouse genome. This allowed us to date the appearance of each ERV family in the human lineage. Here, we again used the branch connecting two consecutive species to date the time when a given ERV family appeared in the human genome. Our analysis yielded identical results to those of Thomas and Schneider (8). For instance, we could not decide with confidence whether 4 different families of ERVs had appeared between basal primates and new world monkeys, or between new world monkeys and old world monkeys.

***Zinc-Finger genes on human chromosome 19:***

Additionally, we observed a higher amount of genetic variation of C2H2 sequences on the q arm of chromosome 19 than on the p arm (see Figure 4). This higher diversity of C2H2 sequences is probably due to a more ancient origin of the zinc fingers located on the q arm. The clusters on the q arm are located on regions of syntenic homology on single chromosomes in chimpanzees (chromosome 19), mice (chromosome 7), and dogs (chromosome 1) (10). This compares with the two major clusters on the p arm, which are syntenic to different regions in more than one chromosome in mice (11). In apes, the clusters on the p arm remain syntenic to regions in single chromosomes (10). Therefore, the configuration of clusters present in the p arm of human chromosome 19 emerged some time after rodent-primate divergence, while the configuration observed on the q arm was present in the common ancestor of boroeutheria (i.e. dog-mouse ancestor). To better estimate the time when the present configuration of clusters on human chromosome 19 appeared, we downloaded from the UCSC genome browser the chain of alignments between human chromosome 19 and regions of syntenic homology in the marmoset genome (calJac3). We found that every cluster of zinc fingers on human chromosome 19 was syntenic to a cluster on chromosome 22 in marmosets. Therefore, the present configuration of C2H2 clusters on a single chromosome (syntenic to human chromosome 19) appeared at some time after rodent-primate divergence and before the split of new world and old world monkeys.

**Prediction of DNA binding affinities associated with tandems of Zinc-Fingers**

In the canonical model for the interaction between C2H2 zinc fingers and DNA, only four amino acid-nucleotide contacts per finger are considered to be relevant in the interaction (5). In particular, the positions −1, 2, 3, and 6 of the α-helix of each finger are assumed to be the only amino acids that interact with DNA. In this model, every possible ZF-DNA configuration can be specified by a vector x**_pna_** (here, **p**=0,1,2,3 denotes the position of the nucleotide-amino acid pair, **n**=A, G, T or C denotes the value of the nucleotide at the position **p** and **a** denotes one of the 20 possible amino acids at **p**), in which either x**_pna_** is 1 when the nucleotide-amino acid pair is **na** at the position **p** or x**_pna_** is 0 otherwise. This yields a feature space of finger-DNA configurations with 4×4×20=320 dimensions.

To estimate the DNA binding affinity of a given tandem of fingers we used a Support Vector Machine (SVM) with a 2nd-degree polynomial as kernel function to separate bound states from non-bound states in this feature space. This SVM was trained by Persikov et al. (6) using a “high-quality literature-derived experimental database of ZF–DNA binding examples.” The software implementation of this SVM has been used in several studies to predict different binding sites that were validated experimentally (e.g. (12)). For our study, we wrote a Python program where we used the binding affinities predicted by this SVM (6) to infer the top 10 motifs on any DNA sequence in which a given tandem is most likely to bind. As the best performance of the SVM occurs with tandems that contain three fingers (6), we restricted our analysis to tandems that only had two, three or four fingers. In the case of polydactyl ZF genes that contained more than 4 fingers, we considered every possible combination of four consecutive fingers and estimated the binding affinity of each of these reduced tandems. This gave rise to a significantly larger number of tandems than the one of ZF genes. In particular, the 712 human ZF genes that we studied contained a total of 4,390 tandems with fewer than five fingers.

In order to find candidate ZF repressors of particular ERVs, we evaluated the binding affinity of every possible motif for every tandem of Zinc Fingers on the 4,700 human retroviral sequences that we had isolated. When comparing the spectrum of bound states of two different tandems, we performed a quantile normalization of the SVM-scores associated with each tandem. Therefore, we compared the binding affinity of two different tandems to the same DNA motif, by means of the quantile-normalized scores associated with each tandem. In addition to searching for repressors of ERVs, we also compared the spectrum of bound states of homologous tandems across species when inferring the time at which particular human tandems gained their current DNA binding affinity (see **Detection of Zinc-Finger domains and families of endogenous retroviruses in primate and rodent genomes**).

**Inference of the preferential location of zinc-finger repressors of ERVs on chromosome 19 using Fisher’s exact test with a noisy classifier**

When the determination of the tandems of zinc finger repressors that recognize motifs specific to ERV families is perfect, the expected null distribution for observing k repressors in chromosome 19 is given by the familiar hypergeometric distribution


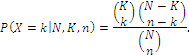


Here, N=4390 denotes the total number of tandems of zinc fingers in the genome, K=2492 is the total number of tandems located in chromosome 19 and n=41 is the total number of tandems identified as targeting ERVs. In our case, we observe k=32 repressors located in chromosome 19, giving rise to a p-value of 0.003.

However, our method of identifying the tandems of zinc fingers that are repressors of particular ERV families is not perfect. Therefore, the probability of misidentifying the location of the repressor cannot be neglected. This is because there is an error associated with the inference of the DNA binding motifs of a given tandem of zinc fingers using a support vector machine (6). We have reduced this error by analyzing only those zinc fingers that gained their binding affinity at the same time that the given ERV invaded the human lineage. However, the error should still remain significant. Nevertheless, we show below that even if the classifier of the chromosomal location has a significant misclassification rate, our inference that the preferential location of the zinc-finger repressors is on chromosome 19 is still statistically significant.

In particular, let *p* be the probability that a repressor being located in chromosome 19 is misclassified as being located elsewhere. Similarly, let *q* be the probability that a repressor being located in a chromosome that is not chromosome 19, is misclassified as being located in chromosome 19. Therefore, if the true number of repressors located in chromosome 19 is α, our noisy classifier will usually find a different number, that we denote as k=γ+β, of repressors located in chromosome 19. The number k=γ+β means that a number of γ repressors were correctly classified as being located in chromosome 19, α-γ repressors were incorrectly classified as being located in other chromosomes, β repressors located in other chromosomes were incorrectly classified as being located in chromosome 19, and n-α-β repressors were correctly classified as being located in other chromosomes. Therefore, given that the true number of repressors located in chromosome 19 is α, the probability of observing k repressors located in chromosome 19 using the noisy classifier is:


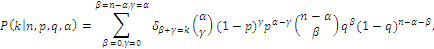


here, δ_γ+β=k_ denotes the Kronecker delta function, which is one for γ+β=k and it is zero otherwise. Therefore, summing over all possible true numbers of repressors located in chromosome 19, gives rise to the null distribution of observing k repressors in chromosome 19:


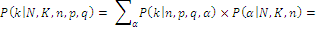


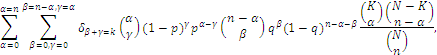


where N is the total number of tandems of zinc fingers everywhere, K is the total number of tandems located in chromosome 19, n is the total number of tandems to be identified as targeting ERVs, and k is the observed number of tandems that target ERVs and that are located in chromosome 19.

In our particular case, we know the values for N, K, n and k (N=4390, K=2492, n=41 and k=32), but we do not know the exact values for *p* and *q*. However, one can show by computing the p-values associated with different *p*‘s and *q*‘s, that any combination of misclassification probabilities with *q*<0.2 gives rise to p-values smaller than 0.05. We can use this to show how our observed bias of the chromosomal locations of zinc finger repressors towards chromosome 19 is significant. Let us denote by F the probability that the SVM predicts a wrong tandem of zinc fingers repressor of a particular ERV. Therefore, the probability *q* of misclassifying the repressor as being falsely located in chromosome 19 is approximately


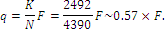


As values of *q* that are smaller than 0.2 give rise to a statistically significant evidence that the repressors are preferentially located on chromosome 19, it is sufficient for the probability F to be less than 0.35. This is consistent with the error rates reported in Persikov et al. (6).

# Works Cited

x

| 1. | The 1000 Genomes Project. A map of human genome variation from population-scale sequencing. Nature. 2010; 467: p. 1061–1073. |
| --- | --- |
| 2. | Gravel S, Henn BM, Gutenkunst RN, Indap AR, Marth GT, Clark AG, et al. Demographic history and rare allele sharing among human populations. PNAS. 2011; 108(29): p. 11983-11988. |
| 3. | Kimura M. Some Problems of Stochastic Processes in Genetics. The Annals of Mathematical Statistics. 1976; 28(4): p. 882-901. |
| 4. | Lukic S, Hey J, Chen K. Non-equilibrium allele frequency spectra via spectral methods. Journal of Theoretical Population Biology. 2011; 79(3): p. 203-219. |
| 5. | Wolfe SA, Nekludova L, Pabo CO. DNA RECOGNITION BY Cys2His2 ZINC FINGER PROTEINS. Annual Review of Biophysics and Biomolecular Structure. 2000; 29 : p. 183-212. |
| 6. | Persikov AV, Osada R, Singh M. Predicting DNA recognition by Cys2His2 zinc finger proteins. Bioinformatics. 2009; 25: p. 22-29. |
| 7. | Tristem M. Identification and Characterization of Novel Human Endogenous Retrovirus Families by Phylogenetic Screening of the Human Genome Mapping Project Database. Journal of Virology. 2000; 74(8). |
| 8. | Thomas J, Schneider S. Coevolution of retroelements and tandem zinc finger genes. Genome Research. 2011; 21: p. 1800-18012. |
| 9. | Wolf D, Goff SP. Embryonic stem cells use ZFP809 to silence retroviral DNAs. Nature. 2009; 458(7242): p. 1201-1204. |
| 10. | Huntley S, Baggott DM, Hamilton AT, Tran-Gyamfi M, Yang S, Kim J, et al. A comprehensive catalog of human KRAB-associated zinc finger genes: Insights into the evolutionary history of a large family of transcriptional repressors. Genome Research. 2006; 16: p. 669-677. |
| 11. | Dehal P, Predki P, Olsen AS, Kobayashi A, Folta P, Lucas S, et al. Human Chromosome 19 and Related Regions in Mouse: Conservative and Lineage-Specific Evolution. Science. 2001; 293(5527): p. 104-111. |
| 12. | Myers S, Bowden R, Tumian A, Bontrop RE, Freeman C, MacFie TS, et al. Drive Against Hotspot Motifs in Primates Implicates the PRDM9 Gene in Meiotic Recombination. Science. 2010; 327(5967): p. 876-879. |

x
